# Influence of mild cognitive impairment on clinical and functional prognosis in older candidates for cardiac surgery

**DOI:** 10.3389/fnhum.2023.1158069

**Published:** 2024-01-11

**Authors:** Magali González-Colaço Harmand, María Mata, Pablo César Prada-Arrondo, Alberto Domínguez-Rodríguez, José Barroso, Ivan Galtier

**Affiliations:** ^1^Department of Internal Medicine-Geriatric Medicine, University Hospital Nuestra Señora de Candelaria, Santa Cruz de Tenerife, Spain; ^2^Faculty of Health Sciences, Universidad Europea de Canarias, La Orotava, Spain; ^3^School of Psychology, University of La Laguna, San Cristóbal de La Laguna, Spain; ^4^Department of Cardiac Surgery, University Hospital of Canary Islands, San Cristóbal de La Laguna, Spain; ^5^Department of Cardiology, University Hospital of Canary Islands, San Cristóbal de La Laguna, Spain; ^6^Department of Psychology, Faculty of Health Sciences, University Fernando Pessoa Canarias, Las Palmas, Spain

**Keywords:** mild cognitive impairment, cardiac surgery, cognitive domains, elderly, prognosis

## Abstract

**Introduction:**

In this study, we analyzed the prognostic impact of mild cognitive impairment (MCI) prior to cardiac surgery on 12-month clinical outcomes in older patients.

**Method:**

We performed a longitudinal prospective study of 48 patients undergoing cardiac surgery and 26 neurologically healthy participants aged 65 years or older. All participants underwent a neuropsychological assessment. Functional status, quality of life and frailty were assessed in candidates for surgery. One year after surgery, 24 patients remained in the study.

**Results:**

Mild cognitive impairment (MCI) was diagnosed in 35% of the patients at baseline. Postsurgical changes in functionality consisted of a tendency toward impaired basic activities of daily living (BADL) in the MCI group and a statistically significant worsening in instrumental activities of daily living (IADL) in women with MCI. Changes in quality of life consisted of a significant improvement in anxiety-depression in the MCI group and a tendency toward greater pain-discomfort in the non-MCI group. Cognitive status significantly declined only in the non-MCI group. Neither group showed significant changes in frailty. Relative risk analysis showed that patients with a diagnosis of MCI at baseline had a higher risk of cognitive decline at follow-up, while those without a diagnosis of MCI at baseline had a lower risk of impaired IADL. No association was found between MCI and resource use. Preoperative impairment in memory, visuospatial and executive functions was significantly associated with loss of quality of life at follow-up. Impairment of memory and visuospatial function was significantly associated with cognitive decline. Preserved memory was associated with a lower risk of impaired BADL at follow-up.

**Conclusion:**

The present study provides clue on the impact of MCI in candidates for cardiac surgery. Preoperative detection of cognitive impairment could be highly valuable to help guide pre- and post-operative management.

## 1 Introduction

Because of the increase in life expectancy in the last few decades, 18% of the population in Spain is currently older than 65 years and this percentage is expected to rise to around 40% in 2064 (Instituto Nacional de Estadística, 2014). This demographic change carries with it an increase in age-related cardiovascular diseases, such as diabetes, hypertension, and hypercholesterolemia. Thus, 25% of patients older than 75 years have symptoms of cardiovascular disease (Castillo et al., 2009). In previous decades, the management of these patients was predominantly medical but—parallel to the better health status of these patients and advances in anesthetic and perioperative techniques—many more are currently candidates for cardiac surgery. Indeed, 40% of patients undergoing cardiac surgery in Europe are older than 75 years (Castillo et al., 2009), with a clear increase in the proportion of very old individuals (older than 80 years), who represent up to 9% of candidates for cardiac surgery (Rosborough, 2006; López-Rodríguez et al., 2008). In Spain, cardiac surgery in older patients represents 30% of clinical activity in these services, with a progressive increase in the last few years (Rodríguez et al., 2002).

However, assessment of surgical risk in this population is inadequate and current assessment scales, EuroScore I and II (Lafuente et al., 2008) and the Society of Thoracic Surgeons (STS) score (The Society of Thoracic Surgeons, 2012) are not calibrated for older patients. These scales erroneously estimate the intra- and post-operative risk of morbidity and mortality in older patients (López-Rodríguez et al., 2008), and, moreover, do not help to determine key parameters in surgical decision-making, such as post-operative functional prognosis and quality of life in these patients. In an attempt to refine prognosis in older patients, some studies have begun to measure clinically relevant parameters in this age group. For example, assessing frailty, understood as a state of greater vulnerability to organ stress, could help to identify which older adults will have higher short- and long-term mortality, longer hospital stay, or a higher complication rate (Lee et al., 2010; Sündermann et al., 2011; The Society of Thoracic Surgeons, 2012). In the assessment of older patients before cardiac surgery, one parameter should be a priority: the presence of preoperative cognitive impairment.

The prevalence of mild cognitive impairment (MCI) (Winblad et al., 2004) in the general population, is 7%, while that of dementia is as high as 13% (Luck et al., 2010). There are few publications on candidates for cardiac surgery with prior dementia, since these patients are usually deemed unsuitable for surgery (only 3% of candidates for cardiac surgery have prior dementia) or are referred for endovascular procedures due to their frailty (Baldasseroni et al., 2020; Emmert et al., 2022). The scarce data available show cognitive decline rates of around 21% in these candidates (Bendikaite and Vimantaite, 2020). However, current studies have not determined the impact of MCI prior to cardiac surgery on morbidity and mortality, functional and cognitive prognosis, or quality of life in older patients undergoing cardiac surgery (Oldham et al., 2015; Itagaki et al., 2020).

Due to the high rates of MCI in older candidates for cardiac surgery, there is a need to assess the prognostic impact of MCI prior to cardiac surgery on 12-month clinical outcomes in terms of functional status, institutionalization, quality of life, changes in frailty, cognitive decline, and mortality.

## 2 Materials and methods

### 2.1 Participants

We performed a longitudinal prospective study in patients who were candidates for major cardiac surgery at the University Hospital of the Canary Islands (HUC). At baseline of the study, there were 48 patients and 26 neurologically healthy participants aged 65 years or older. The exclusion criteria for surgical candidates were the following: (a) referral for urgent or emergent surgery; (b) admission for a surgical reintervention; (c) prior diagnosis of a major psychiatric disorder or intellectual disability; (d) a history of acquired brain damage (traumatic brain injury); (e) substance abuse; and (f) visual and/or hearing impairment that would hamper neuropsychological assessment.

Control subjects were drawn from the population-based sample of the Neuropsychological Studies Group of the Canary Islands (GENIC) (Ferreira et al., 2017). At baseline, patients and controls did not differ in age, sex, educational level, manual preference, mini-mental state examination (MMSE) scores (Folstein et al., 1975), or depression measured by the Geriatric Depression Scale (GDS) (Sheikh and Yesavage, 2008). Exclusion criteria for controls were as follows: (a) a diagnosis of dementia (McKhann et al., 2011) and/or a MMSE score of less than 24 points; (b) a history of vascular disease (cardiac alterations or stroke); (c) a history of cardiovascular risk factors, such as hypertension, diabetes mellitus or dyslipidemia without drug therapy; (d) diagnosis of a major psychiatric disorder; (e) acquired brain damage (traumatic brain injury); (f) a history of substance abuse; and (g) visual and/or hearing impairment that would hamper neurological assessment.

All participants took part in the study voluntarily and signed an informed consent form. This study got the approval from the Research Ethics Committee of Canary Islands University Hospital [protocol code 2017_39 (CogCC)] on 25th May 2017, and was done and in line with the principles of the Declaration of Helsinki for Research in Humans.

### 2.2 Procedure

Patients who were candidates for cardiac surgery were referred for preoperative assessment by a multidisciplinary team composed of a geriatrician and a neuropsychologist. History and physical and neurological examination were performed, as well as a clinical interview that collected data likely to provide information on the presence of cognitive decline. The following tests were also performed:

#### 2.2.1 Neuropsychological assessment

We applied a neuropsychological protocol grouped by cognitive domains. Attention was examined using the Vienna Reaction Test PC-Vienna System (Schuhfried, 1992), an instrument that allows the presentation of visual and auditory targets. The number of correct reactions to target was recorded. Executive functions were assessed by the Phonemic Verbal Fluency Test and the Animal Fluency Test (Benton et al., 1989). In these tasks participants must generate words beginning by a given letter (F, A, S), and generate words that belong to the semantic of animals. The number of words generated correctly for 1 min was recorded. Memory was assessed by the California Verbal Learning Test (Delis et al., 1987), which includes learning over a five-trial presentation of a 16-word list, free and cued delayed recall, and recognition. Visuospatial functions were examined using a simplified version of the Judgment of Line Orientation Test (JLOT, 15-item) (Benton et al., 1983). In this task, participants must make judgments about the spatial orientation of line segments. The number of correct responses was recorded. Language was assessed by a naming test (Druks and Masterson, 2000) consisting of naming 20 pictorial stimuli representing actions. The Blessed Dementia Rating Scale (BDRS) (Blessed et al., 1968) was also applied. This scale has 22 items and assessed the functional abilities and changes in habits and behavior of people with cognitive impairment.

#### 2.2.2 Functional status, quality of life and frailty variables

Functional status was evaluated by scales validated internationally and in Spanish. The Katz scale (Katz et al., 1963) was used to assess functional status in basic activities of daily living (BADL). This scale ranks adequacy of performance in six basic daily living functions. Patients are scored yes/no for independence in each of the six functions. Functional alteration was considered to be present if the participant was unable to perform an activity alone or needed help in more than one item. The Lawton and Brody (1969) scale was used to assess functional status for instrumental activities of daily living (IADL). This scale consists of 5 items for men and 8 items for women. Functional alteration was defined as a score lower than 4 in men and lower than 6 in women. Quality of life was assessed with the EuroQL 5D 5L scale (Oemar and Janssen, 2013), which assesses 5 dimensions independently: mobility, self-care, usual activities, pain/discomfort, and anxiety/depression. Impairment in the various domains was considered to be present if the individual reported problems in performing an activity or physical/psychological distress. Finally, frailty was measured by the Fried frailty phenotype (Fried et al., 2001). The severity of frailty was classified in 3 levels: (1) robust (0 points), (2) prefrail (1 to 2 points) and (3) frail (3 to 5 points). In this study, we dichotomized this variable to reflect the absence (level 1) or presence (level 2 and 3) of frailty.

#### 2.2.3 Diagnosis of mild cognitive impairment and dementia

Diagnosis of MCI was based on the history and neuropsychological examination performed, following the criteria of Winblad et al. (2004). Patients had to have cognitive complaints that evidence of cognitive decline, measured either by self and/or informant report, in conjunction with deficits in objective cognitive tasks. In this regard, the patients had to have alterations in one or more cognitive functions, demonstrated by performance equal to or less than 1.5 standard deviations below the mean of the control subjects. In addition, the patients had to have preserved functionality for BADL, although they could have minimal deterioration for IADL.

Diagnosis of dementia was based on the criteria of McKhann et al. (2011). The patient had to have alterations in at least 2 or more cognitive functions. In addition, diagnosis of MCI or dementia was based on demonstration of significant interference in the ability to function at work or in BADL.

At 12 months post-discharge, the baseline parameters were measured again (clinical, neuropsychological, functional status, quality of life and frailty); we also gathered data on mortality, institutionalization, and healthcare resource use.

### 2.3 Statistical analysis

The Mann–Whitney U test was used for comparisons between patients and the controls at baseline. The Wilcoxon Rank sum test was used for pre-post comparisons of quantitative variables. Relative risk (RR) analysis and the chi-square test were used to analyze the predictive value of MCI, as well as the influence of alterations in the various cognitive domains on functional status, frailty, and quality of life. Independent predictive variables are expressed as RR with 95% confidence intervals (CI). Statistical significance was set at *p* < 0.05. All statistical analyses were performed with SPSS-PC software, version 25.0 for Windows.

## 3 Results

The baseline sociodemographic characteristics of patients and controls are shown in Table 1.

**TABLE 1:** Baseline sociodemographic characteristics of patients and controls.

Variable	Controls (*n* = 26) M (SD)	Patients (*n* = 48) M (SD)
Age (years)	73.42 (3.68)	75.31 (5.15)
Age (range)	65–80	65–85
Gender (men/women)	16/10	28/20^b^
Education (years)	6.81 (2.62)	5.81 (3.88)^a^
Side of onset (% of right)	100	97.9^b^
MMSE	28.12 (1.21)	26.69 (3.42)^a^
Information (WAIS-III)	12.00 (5.42)	10.49 (5.91)^a^
GDS	2.57 (1.91)	2.65 (2.76)^a^

*^a^*Comparisons between groups were not significant.

*^b^*Pearson’s chi-square test was not significant. *n*, number of individuals in each group; M, mean; SD, standard deviation; MMSE, mini-mental state examination; WAIS-III, Wechsler Adult Intelligence Scale third edition; GDS, Geriatric Depression Scale.

The most frequent vascular risk factor prior to surgery was hypertension (85.4%). Hypercholesterolemia was present in 68.8% of the patients, casual alcohol use in 58.3%, and diabetes mellitus in 47.9%. A history of stroke was found in 16.7%, thyroid disease in 12.5% and peripheral arterial disease in 10.4%. Only 2.1% were current smokers.

**FIGURE 1:**
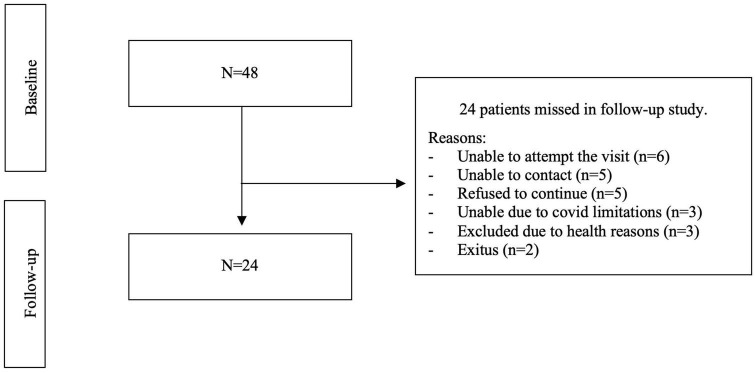
Reasons for discontinuing follow-up.

A total of 24 volunteers participated in the follow-up study. The remaining 24 participants discontinued the study for various reasons (Figure 1).

After completion of the preoperative neuropsychological assessment, cognitive performance was compatible with a diagnosis of MCI in 35.4% of the sample. All patients with MCI had a multidomain profile, i.e., alterations in 2 or more cognitive domains. Cognitive performance was within the normal range (non-MCI) in the remaining 64.6%.

**FIGURE 2:**
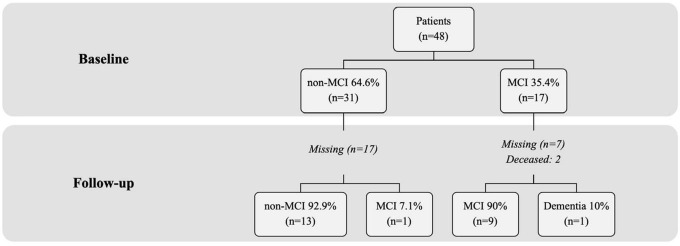
Percentage of patients who developed mild cognitive impairment or dementia. nMCI, patients without mild cognitive impairment; MCI, patients with mild cognitive impairment.

During follow-up, 7.1% (1/14) of non-MCI patients developed MCI, while the remaining 92.9% maintained normal cognitive function. Ten percent (1/10) of patients with MCI at baseline developed dementia, while the remaining 90% maintained MCI (9/10). None of the patients with a diagnosis of MCI at baseline recovered normal cognitive function during follow-up (Figure 2).

The mean post-operative length of stay was 12.69 (SD = 7.82) days, and mean length of stay in the intensive care unit (ICU) was 3.67 (SD = 4.52) days. Readmission to the ICU was required in 15.2% of the patients due to post-operative complications, while 17.4% visited the emergency department after hospital discharge and 2.1% were readmitted to hospital. None of the patients was institutionalized and only 2 patients died.

Pre- and post-operative changes in functional status, quality of life, and frailty in the whole sample are shown in Table 2. There was statistically significant worsening in BADL (*p* = 0.002) and, among the subgroup of women, statistically significant worsening in IADL (*p* = 0.011). In terms of quality of life, mood significantly improved, demonstrated by responses to items on anxiety/depression (*p* = 0.012), while pain/discomfort worsened (*p* = 0.033). No changes were found in frailty or on the BDRS.

**TABLE 2:** Changes in quality of life, functionality, frailty, and cognitive impairment.

	Baseline (*n* = 48) M (SD)	Follow-up (*n* = 24) M (SD)	*p*-values
**QoL**			
Mobility	2.04 (0.74)	1.86 (0.94)	0.518
Self-care	1.13 (0.33)	1.32 (0.89)	0.257
Usual activities	1.29 (0.58)	1.50 (0.74)	0.096
Pain/discomfort	1.77 (0.72)	2.05 (0.79)	0.033
Anxiety/depression	2.04 (0.82)	1.32 (0.57)	0.012
**BADL**	1.27 (0.92)	1.58 (0.72)	0.002
**IADL**			
Men	4.89 (0.42)	4.52 (1.08)	0.141
Women	6.65 (2.32)	4.55 (3.17)	0.011
**Frailty**	1.92 (1.46)	1.52 (1.23)	0.206
**BDRS**	2.01 (2.25)	1.32 (2.73)	0.167

*n*, number of patients in each group; M, mean; SD, standard deviation; QoL, quality of life; BADL, basic activities of daily living; IADL, instrumental activities of daily living; BDRS, Blessed Dementia Rating Scale.

Pre- and post-operative changes in functionality, quality of life and frailty, depending on preoperative cognitive status, are shown in Table 3. Anxiety/depression significantly improved (*p* = 0.034) in patients with MCI at baseline. BADL showed a tendency to worsen in patients with MCI (*p* = 0.059), while autonomy for IADL significantly worsened in the group of women with MCI (*p* = 0.039). The non-MCI group showed worsening functionality in BADL (*p* = 0.011) and a decline in cognitive status (*p* = 0.031). The non-MCI group also showed a tendency toward worsening of pain/discomfort (*p* = 0.052). Neither group showed significant changes in frailty.

**TABLE 3:** Changes in quality of life, functionality, frailty, and cognitive impairment in diagnostic groups at baseline.

	MCI (*n* = 17)			nMCI (*n* = 31)		
	Baseline M (SD)	Follow-up M (SD)	*p*-values	Baseline M (SD)	Follow-up M (SD)	*p*-values
**QoL**						
Mobility	2.06 (0.66)	2.00 (1.05)	0.454	2.03 (0.80)	1.75 (0.87)	> 0.99
Self-care	1.24 (0.44)	1.60 (1.26)	0.180	1.06 (0.25)	1.08 (2.89)	> 0.99
Usual activities	1.29 (0.47)	1.70 (0.95)	0.180	1.29 (0.64)	1.33 (0.49)	0.317
Pain/discomfort	1.76 (0.75)	2.00 (0.94)	0.317	1.77 (0.72)	2.08 (0.67)	0.052
Anxiety/depression	2.18 (0.81)	1.40 (0.52)	0.034	1.97 (0.834)	1.25 (0.62)	0.160
**BADL**	1.53 (1.46)	1.67 (0.89)	0.059	1.13 (0.34)	1.53 (0.61)	0.011
**IADL**						
Men	4.75 (0.71)	4.20 (1.10)	0.317	4.95 (0.22)	4.63 (1.09)	0.276
Women	5.56 (3.09)	4.71 (3.09)	0.039	7.55 (0.82)	4.25 (3.77)	0.109
**Frailty**	2.12 (1.54)	1.90 (0.99)	0.163	1.81 (1.42)	1.27 (1.33)	0.558
**BDRS**	2.53 (3.05)	2.47 (3.70)	0.673	1.73 (1.65)	0.72 (1.89)	0.031

*n*, number of the samples in each group; MCI, group of patients with mild cognitive impairment at baseline; nMCI, group of patients without mild cognitive impairment at baseline; M, mean; SD, standard deviation; QoL, quality of life; BADL, basic activities of daily living; IADL, instrumental activities of daily living; BDRS, Blessed Dementia Rating Scale.

The relative risk (RR) analysis and the chi-square test were used as an alternative approach to analyze the predictive value of MCI (Table 4). The non-MCI group had a lower risk of impaired IADL, while the MCI group had a higher risk of decline on the BDRS. No association was found between MCI and resource use (ICU stay, hospital stay, ICU readmission, hospital readmission, or emergency department visits during follow-up).

**TABLE 4:** MCI at baseline as predictor of quality of life, functionality, frailty, and cognitive impairment at baseline and follow-up.

Baseline							Follow-up					
	MCI (*n* = 17)	nMCI (*n* = 31)	RR	95% IC	*X* ^2^	*p*	MCI (*n* = 10)	nMCI (*n* = 14)	RR	95% IC	*X* ^2^	*p*
	*n* (%)	*n* (%)					*n* (%)	*n* (%)				
**Altered QoL**												
**Mobility**												
Yes	14/17 (82.4)	22/31 (71)	1.160	0.847–1.590	0.759	0.384	6/9 (66.7)	6/12 (50)	1.333	0.642–2.768	0.583	0.445
No	3/17 (17.6)	9/31 (29)					3/9 (33.3)	6/12 (50)				
**Self-care**												
Yes	4/17 (23.5)	2/31 (6.5)	3.647	0.743–17.902	2.928	0.087	3/9 (33.33)	1/12 (8.3)	4.000	0.494–32.393	2.085	0.149
No	13/17 (76.5)	29/31 (93.5)					6/9 (66.7)	11/12 (91.7)				
**Usual activities**												
Yes	5/17 (29.4)	6/31 (19.4)	1.520	0.543–4.252	0.629	0.428	5/9 (55.6)	4/12 (33.3)	1.667	0.619–4.489	1.037	0.309
No	12/17 (70.6)	25/31 (80.6)					4/9 (44.4)	8/12 (66.7)				
**Pain/discomfort**												
Yes	10/17 (58.8)	19/31 (61.3)	0.960	0.590–1.561	0.028	0.867	6/9 (66.7)	10/12 (83.3)	0.800	0.472–1.355	0.787	0.375
No	7/17 (41.2)	12/31 (38.7)					3/9 (33.3)	2/12 (16.7)				
**Anxiety/depression**												
Yes	14/17 (82.4)	20/31 (64.5)	1.276	0.907–1.796	1.691	0.194	4/9 (44.4)	2/12 (16.7)	2.667	0.619–11.493	1.944	0.163
No	3/17 (17.6)	11/31 (35.5)					5/9 (55.6)	10/12 (83.3)				
**BADL altered**												
Yes	1/17 (5.9)	0/31 (0)					1/9 (11.1)	0/13 (0)				
No	16/17 (94.1)	31/31 (100)	0.941	0.836–1.060	1.862	0.172	8/9 (88.9)	13/13 (100)	0.889	0.706–1.120	1.513	0.219
**IADL altered**												
Yes	4/17 (23.5)	0/31 (0)					4/9 (44.4)	2/13 (15.4)	2.889	0.665–12.555	2.264	0.132
No	13/17 (76.5)	31/31 (100)	0.765	0.587–0.995	7.957	0.005	5/9 (55.6)	11/13 (84.6)				
**Frailty altered**												
Yes	14/17 (82.4)	25/31 (80.6)	1.021	0.772–1.351	0.021	0.885	8/9 (88.9)	8/13 (61.5)	1.444	0.887–2.353	2.006	0.157
No	3/17 (17.6)	6/31 (19.4)					1/9 (11.1)	5/13 (38.5)				
**Altered BDRS**												
Yes	5/17 (29.4)	5/31 (16.1)	1.824	0.613–5.420	1.174	0.278	5/10 (50)	2/14 (14.30)	3.500	0.842–14.552	3.601	0.058
No	12/17 (70.6)	26/31 (83.9)					5/10 (50)	12/14 (85.7)				

*n*, number of patients in each group; QoL, quality of life; BADL, basic activities of daily living; IADL, instrumental activities of daily living; BDRS, Blessed Dementia Rating Scale.

**FIGURE 3:**
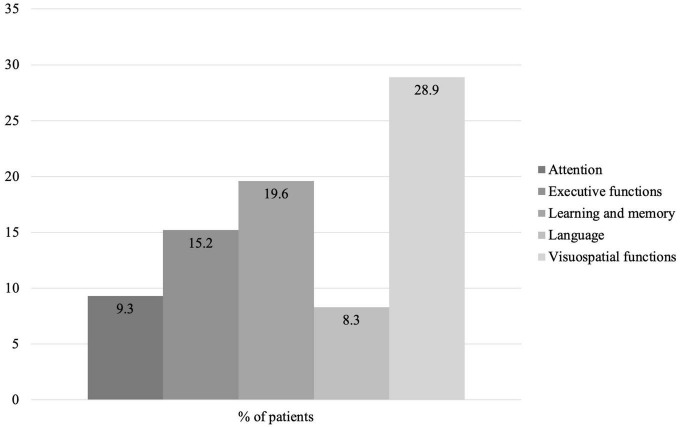
Percentage of patients with impairment by cognitive domain at baseline.

We decided to analyze baseline decline in cognitive domains, independently of diagnosis, as a predictor of impaired quality of life, functionality, frailty, and cognitive status. The most frequently altered cognitive domains in the group of preoperative patients are shown in Figure 3.

The cognitive domains showing predictive value in the preoperative results of clinical variables were language, visuospatial functions, and executive functions. Language impairment was significantly associated with impaired quality of life (self-care) (RR = 11.000, 95% CI = 3.215–37.640, *X*^2^ = 15.584, *p* = 0.000) and lesser autonomy in IADL (RR = 11.000, 95% CI = 2.068–58.519, *X*^2^ = 9.917, *p* = 0.002). Language preservation was significantly associated with a lower risk of impaired BADL (RR = 0.750, 95% CI = 0.426–1.321, *X*^2^ = 11.234, *p* = 0.001). Preservation of visuospatial functions was significantly associated with a lower risk of impairment in IADL (RR = 0.846, 95% CI = 0.671–1.067, *X*^2^ = 5.152, *p* = 0.023). Alterations in executive functions were related to decline in IADL (RR = 11.143, 95% CI = 1.157–199.633, *X*^2^ = 6.584, *p* = 0.010).

The cognitive domains showing predictive power for the post-operative results of clinical variables were memory, visuospatial functions, and executive functions. Memory impairment was significantly associated with impaired quality of life (mood) (RR = 4.750, 95% CI = 1.989–11.346, *X*^2^ = 5.526, *p* = 0.019) and with cognitive decline (BDRS) (RR = 4.400, 95% CI = 0.2036–9.508, *X*^2^ = 5.299, *p* = 0.021). Memory preservation was significantly associated with a lower risk of impairment in BADL (RR = 0.500, 95% CI = 0.125–1.999, *X*^2^ = 10.476, *p* = 0.001). Impaired visuospatial functions were significantly associated with loss of quality of life (self-care) (RR = 6.000, 95% CI = 0.755–47.708, *X*^2^ = 3.860, *p* = 0.049), and showed a tendency toward risk of cognitive decline (BDRS) (RR = 3.750, 95% CI = 0.867–16.220, *X*^2^ = 3.638, *p* = 0.056). Impaired executive functions were associated with loss of quality of life (pain/discomfort), but this finding was not statistically significant (RR = 0.400, 95% CI = 0.080–2.008, *X*^2^ = 3.544, *p* = 0.060). No association was found between the remaining cognitive domains and the clinical variables.

## 4 Discussion

The results showed that the prevalence of MCI in our sample was higher than those reported in studies on cognitive decline in the literature, with rates above 35% (Bendikaite and Vimantaite, 2020). This could be because most prior studies did not conduct a broad neuropsychological assessment for diagnosis of MCI and used only screening tests, such as the MMSE (Veliz-Reissmüller et al., 2007; Bendikaite and Vimantaite, 2020; Itagaki et al., 2020). Only a few publications used more exhaustive diagnostic criteria with batteries of neuropsychological tests (Oldham et al., 2015).

A notable finding was the absence of patients in our sample with dementia at baseline. This could be because, since the implantation of endovascular procedures such as transcatheter aortic valve implantation (TAVI), patients with frailty or poor clinical status have been referred mainly for this type of procedure. In some studies, pre-TAVI dementia rates have exceeded 4% (Park et al., 2022). Equally, in line with findings in the literature (Knipp et al., 2017; Whitlock et al., 2019; Bendikaite and Vimantaite, 2020), we found no evidence that patients developed dementia, despite a decline in their cognitive performance.

The results of the present study concur with those in the literature on overall worsening of functional status (Hiltrop et al., 2016; Miguelena-Hycka et al., 2019; Prada-Arrondo, 2021). In previous studies, functional outcomes were worse in patients with a diagnosis of cognitive decline (Bendikaite and Vimantaite, 2020). However, in our study, post-operative functional deterioration was more pronounced in cognitively healthy patients, possibly because patients with MCI had greater preoperative functional decline, making the change less evident.

Unlike previous studies (Bendikaite and Vimantaite, 2020), this functional decline was accompanied by an improvement in post-operative quality of life, associated with a reduction in anxiety/depression in patients with cognitive decline. These results are especially important because mood is known to be altered in patients prior to cardiac surgery (by up to 30% in some series), due to fear of the intervention and the debilitating effects of the cardiac symptoms (Connerney et al., 2001; Horne et al., 2013). In general, these mood alterations usually persist or even worsen after surgery rather than improving, as in our sample. Therefore, these results represent a true geriatric paradox, since quality of life is usually determined by functional status in older patients (Darnton-Hill, 1995), and can only be explained by the relief experienced by these patients after the intervention, independently of their functional status.

The present study shows the value of MCI in the prognosis of post-operative cognitive status. A diagnosis of MCI was associated with a higher risk of cognitive decline, assessed by the BDRS. A notable finding was the lack of association between MCI and frailty (Kelaiditi et al., 2013; Li et al., 2019), an association that has been previously reported, and that in the last few decades has sparked debate on the diagnostic criteria of cognitive frailty and its relationship with cardiovascular risk factors and cardiovascular disease. In our sample, frailty did not change, even in patients without cognitive changes, which may be due to the small sample size, since other studies have reported that frailty improved after surgery (Miguelena-Hycka et al., 2022).

To gain deeper insight into these results, we analyzed the relationship between impaired cognitive domains at baseline and the studied outcomes. We found that a heterogeneous pattern of cognitive impairment was associated of a risk of worsening of functional autonomy and quality of life. In this regard, the domains that were significantly associated with pre- and post-operative outcomes were visuospatial functions, memory, and executive functions. These results could be interpreted as reflecting the wide variability in the profile of cognitive involvement in our sample, as indicated by the fact that all patients with a diagnosis of MCI showed impairment in 2 or more cognitive domains and that visuospatial functions, memory and executive functions were the 3 most frequently affected domains (between 15 and 30%). This heterogeneity in the profile of cognitive involvement is consistent with the findings of previous studies, in which the profile of candidates for cardiac surgery was characterized by alterations in more than 2 cognitive domains, especially executive functions, memory, attention, processing speed, and working memory (Vingerhoets et al., 1997; Rankin et al., 2003; Hogue et al., 2006; Hudetz et al., 2012). To a lesser extent, previous studies have also reported involvement in visuospatial functions (Rankin et al., 2003; Hogue et al., 2006) and in some language components, such as naming (Rankin et al., 2003).

Moreover, this heterogeneous pattern of cognitive impairment in candidates for cardiac surgery could reflect the spread of vascular injuries in the neural substrate. In this regard, Lee et al. (2018) found a significant relationship between an elevation of right atrial pressure in chronic valvular heart disease and a greater volume of white matter hyperintensities (WMH). In addition, a recent study found an association between worse cardiovascular health in old age and a greater risk of increased volume of WMH, subcortical vascular injuries, and cortical infarcts (Sedaghat et al., 2023). Similar results have been reported in other vascular diseases, such as carotid stenosis. This entity has been related to reduced cerebral blood flow, a higher incidence of lacunar infarcts and WMH, which mainly affects the border areas of the cerebral arteries (Cheng et al., 2012; Lin et al., 2014). This cerebrovascular damage in the neuronal substrate has been associated with an increased risk of developing dementia (Debette and Markus, 2010). In a longitudinal study, Bombois et al. (2008) found a significant relationship between a large amount of subcortical WMH at baseline and a higher risk of developing vascular or mixed dementia. A recent study by Alban et al. (2023) reported an association between the incidence of WMH and β-amyloid accumulation, which is a biomarker for the development of dementia due to Alzheimer’s disease. Therefore, the results of previous investigations suggest that patients with cardiovascular disease could be at higher risk of developing dementia due to Alzheimer’s disease, vascular dementia, or mixed dementia.

In view of the high prevalence of MCI in our sample, the most important question is whether assessment of cognitive impairment should be included in the preoperative assessment of candidates for cardiac surgery. Despite the general recommendations in forums on cardiac surgery (Murkin et al., 1995), current preoperative assessment protocols do not systematically include evaluation of cognitive decline (prognostic algorithms for the presence of MCI are not included in the STS Score or in Euroscore I and II); equally, assessment of cognitive status is not included in the European guidelines on the management of the main heart diseases used to the define surgical indication for these diseases (Visseren et al., 2021). In their guidelines on the management of patients with valvular heart disease (Fried et al., 2001), the American Heart Association and the American College of Cardiology recommend assessing surgical risk by combining the risk estimate provided by the STS, the presence of major organ dysfunction, the contraindications to each procedure, and frailty–assessed by the Katz index–, and independent ambulation. Other authors recommend that frailty assessment be implemented and accompanied by cognitive status screening, due to its relationship with post-operative delirium, worse recovery, and longer hospital stay (Pérez Vela et al., 2020).

An argument in favor of including cognitive screening is that all candidates for cardiac surgery should undergo comprehensive geriatric assessment (CGA) encompassing functional status, the patient’s social context, and cognitive status. CGA allows personalized adaptation of each patient’s care plan. In our sample, knowing that MCI impairs the patient’s ability to perform IADL may help to stress post-operative maintenance and optimization of these activities. Conversely, paying attention to performance in cognitive functions before surgery, with the aim of improving cognitive status, could help to maintain functional independence. Moreover, including cognitive assessment in the patient history is important as a diagnostic datum to be reported to the primary care team and the patient, with a view to mid-term follow-up of progression to dementia.

The main limitation of this study is its sample size. Because of the small number of events, we were unable to determine the impact of MCI on mortality or institutionalization. Also, we had an important drop-out rate, which would have had less impact if the sample size had been larger.

The study also has several strengths: first, the longitudinal study included only older patients, unlike other studies on cognitive decline (Hovens et al., 2012); second, we used a complete battery of neuropsychological tests, including a clinical interview to obtain data on the functional repercussions of MCI; third, the follow-up lasted 12 months and included all types of major cardiac surgical disease. Consequently, despite its small sample size, this study provides data of interest on the impact of MCI in patients who are candidates for cardiac surgery.

In conclusion, the MCI rate in candidates for cardiac surgery was high and was related to a loss of autonomy in IADL. Quality of life improved in patients with MCI, despite the higher risk of cognitive decline, assessed by the BDRS. We found no relationship between MCI with institutionalization or mortality, due to the absence of patients being transferred to a nursing home or dying during follow-up. We found no relationship between MCI and frailty. Consequently, preoperative detection of MCI could provide data to help guide pre- and post-operative management. In future research, larger sample sizes would be needed to confirm or refute our results. Moreover, neuroimaging should be included to allow correlation between clinical data and objective changes in brain imaging.

## Data availability statement

The raw data supporting the conclusions of this article will be made available by the authors, without undue reservation.

## Ethics statement

The studies involving humans were approved by the Research Ethics Committee of Canary Islands University Hospital [protocol code 2017_39 (CogCC)] on 25th May 2017. The studies were conducted in accordance with the local legislation and institutional requirements. The participants provided their written informed consent to participate in this study.

## Author contributions

MG-CH, PP-A, AD-R, JB, and IG contributed to conception and design of the study. MG-CH, MM, JB, and IG organized the database. MM and IG performed the statistical analysis. MG-CH and MM wrote the first draft of the manuscript. MG-CH, MM, and IG wrote sections of the manuscript. All authors contributed to manuscript revision, read, and approved the submitted version.
